# Comparative analysis of breast and colorectal tumours uncouples loss of DNA methylation in cancer from association with the nuclear periphery

**DOI:** 10.1186/1868-7083-5-S1-S8

**Published:** 2013-08-19

**Authors:** Elizabeth Kerr, Richard R Meehan, Wendy A Bickmore, Duncan Sproul

**Affiliations:** 1 MRC Human Genetics Unit, MRC IGMM, University of Edinburgh, Western General Hospital, Crewe Road, Edinburgh, EH4 2XU, UK

## 

The most significant epigenetic alteration found in cancer cells is a global loss of DNA methylation. Recent analyses of cancer methylomes have suggested that methylation is specifically lost from Lamin-associated domains (LADs) which are associated with the nuclear periphery. This suggests that nuclear organisation is a key influence on epigenetic alterations in cancer but the relationship between nuclear organisation and demethylation has not been directly investigated.

We have combined analyses of cancer methylomes with fluorescence *in-situ* hybridisation (FISH) to determine how nuclear organisation in normal cells relates to alterations in DNA methylation levels in cancer.

Our analyses demonstrate that DNA methylation is lost from different regions of the genome in breast and colorectal tumours. Surprisingly, we find that the propensity to become demethylated in cancer is not correlated with localisation to the nuclear periphery in the corresponding normal cell types. While regions some breast cancer hypomethylated regions are localised to the periphery in normal breast epithelial cells, an equal proportion are found in the nuclear interior.

Our observations decouple hypomethylation in cancer from a peripheral nuclear localisation and suggest that association with the nuclear lamina is not mechanistically involved in the loss of methylation in cancer.

